# Pathologic Th1–Treg Cells Exacerbate Acute Lung Injury and Lethality in Sepsis

**DOI:** 10.3390/cells15060521

**Published:** 2026-03-14

**Authors:** Takuya Murao, Atsushi Murao, Monowar Aziz, Ping Wang

**Affiliations:** 1 Center for Immunology and Inflammation, The Feinstein Institutes for Medical Research, Manhasset, NY 11030, USA; 2 Departments of Surgery and Molecular Medicine, Zucker School of Medicine, Manhasset, NY 11030, USA

**Keywords:** Th1-Treg cells, eCIRP, TLR4, STAT1, STAT5, ALI, sepsis

## Abstract

**Highlights:**

**What are the main findings?**

- eCIRP induces Th1-Treg cells via the TLR4-STAT1/STAT5 signaling axis in sepsis.
- Th1-Treg cells aggravate acute lung injury and mortality in sepsis.

**What are the implications of the main findings?**

- The findings provide novel mechanistic insight into sepsis by defining the pathogenic role of an underappreciated T cell subset.
- Targeting pathogenic Th1-Treg cells potentially alleviates sepsis-induced acute lung injury.

**Abstract:**

Sepsis is characterized by dysregulated immune responses induced by damage-associated molecular patterns, such as extracellular cold-inducible RNA-binding protein (eCIRP), that frequently lead to acute lung injury (ALI) and high mortality. Recently, a subset of CD4^+^ T cells possessing both T helper 1 (Th1) and regulatory T cell (Treg) phenotypes, termed Th1-Treg cells, has been identified; however, their function in sepsis remains unknown. In this study, we investigated the dynamics, induction mechanisms, and functional roles of Th1-Treg cells in the development of sepsis-induced ALI. Polymicrobial sepsis was induced in mice using cecal ligation and puncture. In vivo, Th1-Treg cell accumulation in the lungs was analyzed in WT and CIRP^−/−^ mice following sepsis. In vitro, isolated CD4^+^ T cells from WT and TLR4^−/−^ mice were treated with eCIRP to evaluate Th1-Treg cell differentiation and downstream signaling pathways. STAT1 and STAT5 activation were evaluated, and pharmacological inhibitors were used to assess their involvement. Adoptive transfer of Th1-Treg cells was conducted to determine their functional impact on ALI and mortality in septic mice. We observed a significant accumulation of Th1-Treg cells in the lungs of WT septic mice compared to sham mice. eCIRP drove the induction of Th1-Treg cells in vitro, and CIRP^−/−^ mice exhibited decreased Th1-Treg cell accumulation in the lungs compared to WT mice after sepsis. In parallel to Th1-Treg cell induction, eCIRP activated signal transducer and activator of transcription, STAT1 and STAT5. Both the induction of Th1-Treg cells and the activation of STAT1/5 proteins were significantly attenuated in TLR4^−/−^ mice. Furthermore, pharmacological inhibition of STAT1/5 signaling significantly reduced eCIRP-induced Th1-Treg cell differentiation. Intriguingly, adoptive transfer of Th1-Treg cells significantly exacerbated ALI, resulting in increased mortality in sepsis. Our findings indicate Th1-Treg cells induced by the eCIRP–TLR4–STAT1/5 axis aggravate ALI, worsening mortality in sepsis. Targeting these pathogenic cells potentially alleviates sepsis-induced ALI.

## 1. Introduction

Sepsis is defined as life-threatening organ dysfunction resulting from a dysregulated host response to infection [1]. Sepsis is a major global medical problem, affecting over 50 million people and resulting in approximately 11 million deaths annually worldwide [1,2]. The pathogenesis of sepsis is multifactorial, involving uncontrolled systemic inflammation, immune dysregulation, coagulopathy, and metabolic disturbances [3]. Together, these factors contribute to multiple organ failure. Among the organs affected, the lungs are particularly vulnerable, often representing the earliest site of injury [4]. It has been documented that 25–50% of septic patients develop acute lung injury (ALI), a condition associated with high morbidity and a mortality rate of up to 40% [5]. Despite the advances in supportive care, effective immunomodulatory therapies for sepsis remain limited, highlighting the urgent need to better understand the mechanisms underlying immune dysregulation in this condition.

CD4^+^ T cells are a heterogeneous population of T lymphocytes that play central roles in immune responses [6]. Upon activation, CD4^+^ T cells have the capacity to differentiate into multiple functional subsets [7]. Each of these subsets is distinguished by distinct patterns of surface molecule expression, cytokine production, and lineage-defining transcription factors [8]. Notable subsets of CD4^+^ helper T cells include Th1, Th2, and Th17 cells, which produce distinct sets of cytokines [9,10]. Th1 cells produce pro-inflammatory cytokines, most notably interferon γ (IFNγ), which contributes to inflammatory disorders [11]. There is a suppressive subset of CD4^+^ T cells, known as regulatory T (Treg) cells, that maintain immune homeostasis and restrain inflammation [12]. Treg cells are characterized by the expression of the master transcription factor forkhead box protein P3 (Foxp3) in the nucleus and CD25 (IL-2 receptor α chain) on the cell surface [13,14]. Recent studies have identified distinct populations of CD4^+^ T cells that cannot be fully explained by the classical effector T cell or Treg classifications. A new CD4^+^ T cell subset that possesses both Th1 and Treg phenotypes, represented by IFNγ and Foxp3 expression, respectively, has been recently discovered [15]. This subpopulation is called Th1-Treg cells and is implicated in the pathogenesis of inflammatory bowel disease (IBD), where it contributes to tissue inflammation [16]. A recent study has shown that platelet factor 4 serves as a driver of Th1-Treg cells in the tumor microenvironment [17]. However, the presence, dynamics, function, and induction mechanisms of Th1-Treg cells during sepsis remain unexplored.

The pathophysiology of sepsis has been directly linked to both pathogens and host-derived molecules known as damage-associated molecular patterns (DAMPs). Cold-inducible RNA-binding protein (CIRP) is an 18-kDa RNA chaperone primarily sequestered in the nucleus [18]. Once CIRP is released into circulation, extracellular CIRP (eCIRP) serves as a DAMP to activate several immune cells, such as macrophages, neutrophils, and T cells, to promote their pro-inflammatory response by binding to Toll-like receptor 4 (TLR4) [18]. During sepsis, increased eCIRP levels were observed in the circulation of patients, as well as in experimental animal models [19]. eCIRP has been demonstrated to drive pathogenic polarization in innate immune cells, such as macrophages and neutrophils, during sepsis [18]. For instance, eCIRP induces macrophage polarization via STAT3, a member of signal transducer and activator of transcription (STAT) proteins, which regulate cell differentiation [20]. However, the effects of eCIRP on CD4^+^ T cell subsets, including the potential for the induction of Th1-Treg cells, have not been elucidated.

In this study, we investigated the dynamics of Th1-Treg cells during sepsis and found that these cells are markedly increased in the lungs. We further explored the mechanisms underlying their induction and demonstrated that eCIRP drives the generation of Th1-Treg cells via activation of the TLR4–STAT1/5 signaling axis. The accumulation of Th1-Treg cells exacerbates ALI by promoting inflammation and cell death, ultimately contributing to worsened mortality in sepsis. These findings reveal a previously unrecognized role of Th1-Treg cells as pathogenic mediators in septic lung injury and identify the eCIRP–TLR4–STAT1/5 pathway as a critical mechanism driving their induction.

## 2. Materials and Methods

### 2.1. Animals

Male 8–12-week-old wild-type (WT) C57BL/6 mice were purchased from Charles River Laboratory (Charles River, Wilmington, MA, USA). CIRP^−/−^ mice on the C57BL/6 background were originally gifted from Prof. Jun Fujita (Kyoto University, Kyoto, Japan), and TLR4^−/−^ mice on the C57BL/6 background were obtained from Dr. Kevin Tracey (The Feinstein Institutes for Medical Research, Manhasset, NY, USA). The mice were housed in a temperature-controlled room under a 12-hour (h) light/dark cycle and provided with standard laboratory chow and water. All experiments were performed in accordance with the National Institutes of Health guidelines for the care and use of laboratory animals and were approved by the Institutional Animal Care and Use Committees (IACUC) of The Feinstein Institutes for Medical Research.

### 2.2. Isolation of CD4^+^ T Cells

The spleens were harvested from the 8–12-week-old male WT or TLR4^−/−^ mice. The spleens were minced and filtered through a sterile 70 µm cell strainer (Corning, Corning, NY, USA). The erythrocytes were removed using BD Pharm Lyse^TM^ Lysing Buffer (Cat. No.: 555899, BD Science, Franklin Lakes, NJ, USA), and the cells were washed. Then, CD4^+^ T cells were isolated from the cell suspension using an EasySep^TM^ Mouse CD4^+^ T cell Isolation Kit (Cat. No.: 19502A, STEMCELL, Vancouver, BC, Canada) according to the manufacturer’s instructions. Naïve CD4^+^ T cells were isolated using an EasySep^TM^ Mouse Naïve CD4^+^ T cell Isolation Kit (Cat. No.: 19765A, STEMCELL) according to the manufacturer’s instructions.

### 2.3. Treatment of CD4^+^ T Cells with eCIRP

Isolated CD4^+^ T cells from the WT or TLR4^−/−^ mice were cultured in Roswell Park Memorial Institute (RPMI) 1640 medium (Cat. No.: 11875093, Thermo Fisher Scientific, Waltham, MA, USA), supplemented with 10% fetal bovine serum (FBS), 100 U/mL Penicillin and 100 μg/mL Streptomycin, 1% glutamine and 50 μM 2-mercaptaethanol in a 37 °C incubator under humidified conditions containing 5% CO_2_. Recombinant mouse CIRP (eCIRP) was prepared in-house, and quality control assays were performed, as previously described [21]. CD4^+^ T cells were treated with 1.0, 2.5 µg/mL of eCIRP for the indicated time periods on CD3/CD28 coated plates (Ultra-LEAF Purified anti-mouse CD3ε, Cat. No.: 100340; Ultra-LEAF Purified anti-mouse CD28, Cat. No.: 102116, Biolegend, San Diego, CA, USA) in the presence and absence of 1 µM of STAT1 inhibitor (Fludarabine; Cat. No.: HY-B0069, MedChemExpress, Monmouth Junction, NJ, USA) and/or 10 μM of STAT5 inhibitor (Cat. No.: HY-101853, MedChemExpress), followed by stimulation with Cell Activation Cocktail (with Brefeldin A) (Cat. No.: 423304, Biolegend) for 4 h for cytokine evaluation.

### 2.4. Mouse Model of Sepsis

Intra-abdominal sepsis was induced in the 8–12-week-old male WT or CIRP^−/−^ mice by cecal ligation and puncture (CLP) [21,22]. Briefly, the mice were anesthetized with isoflurane, and a midline abdominal incision was made. The cecum was ligated with a 4-0 silk suture 1 cm proximal to its distal extremity, punctured twice (through and through) with a 22 G needle for the 20 h study and once for the survival study. Sham animals underwent laparotomy without CLP. Following the surgery, 1 mL of saline was injected subcutaneously to prevent surgery-induced dehydration, and 0.05 mg/kg buprenorphine was injected subcutaneously as an analgesic. The lungs were harvested 20 h after the surgery. In the survival study, 0.5 mg/kg BW of Imipenem was administered subcutaneously. The mice were monitored for up to 10 days for the survival studies. The mice were euthanized when they met the criteria for humane endpoints approved by the Institutional Animal Care and Use Committee, including failure to eat or drink, grimace score of 2, hunched posture, immobility, labored breathing, non-responsiveness to touch, and reduced response to human touch.

### 2.5. Isolation of Lung Cells

Lung tissues were minced into small pieces and incubated with 1 mg/mL collagenase type I (Cat. No. LS004197, Worthington Biochemical, Lakewood, NJ, USA) at 37 °C for 30 min with periodic agitation. The digested tissue was then mechanically dissociated, passed through a 70 μm cell strainer (Corning, Corning, NY, USA) and treated with BD Pharm Lyse^TM^ Lysing Buffer before cell collection [23]. The isolated cells were stimulated with Cell Activation Cocktail (with Brefeldin A) for 4 h in RPMI 1640 medium, supplemented with 10% fetal bovine serum (FBS), 100 U/mL Penicillin and 100 μg/mL Streptomycin, and 1% glutamine in a 37 °C incubator under humidified conditions containing 5% CO_2_, after which they were stained with antibodies and analyzed using flow cytometry. Brefeldin A was used to inhibit IFNγ secretion feasible for intracellular staining of IFNγ.

### 2.6. Flow Cytometry

Cell suspensions were incubated with a combination of monoclonal fluorescently conjugated antibodies (Abs): FITC anti-mouse CD4 (Cat. No.: 100510, Biolegend), APC anti-mouse CD25 (Cat. No.: 113709, Biolegend), PerCP/Cyanine5.5 anti-mouse CD183 (CXCR3) (Cat. No.: 126514, Biolegend), PE anti-mouse FOXP3 (Cat. No.: 126404, Biolegend), Brilliant Violet 421 anti-mouse IFNγ (Cat. No.: 505830, Biolegend), PE anti-STAT1 Phospho (Ser727) (Cat. No.: 686404, Biolegend), APC anti-STAT5 Phospho (Tyr694) (Cat. No.: 936906, Biolegend), PE Rat IgG2b, κ Isotype Ctrl (Cat. No.: 400636, Biolegend), and Brilliant Violet 421 Rat IgG1, κ Isotype Ctrl (Cat. No.: 400430, Biolegend). To analyze the intracellular expression of IFNγ and Foxp3, the Foxp3 Staining Buffer Set (Cat. No.: 00-5523-00, Thermo Fisher) was used according to the manufacturer’s instructions. To analyze the intracellular expression of phosphorylated (p) STAT1 and pSTAT5, True-Phos^TM^ Perm Buffer (Cat. N0.: 425401, Biolegend) was used according to the manufacturer’s instructions. TrueStain FcX PLUS (Cat. No.: 156604, Biolegend) was used to prevent nonspecific antibody binding, and cell viability was determined using a Zombie NIR Fixable Viability Kit (Cat. No.: 4231006, Biolegend). Flow cytometric analysis was performed on a BD LSRFortessa Cell Analyzer (BD Biosciences, San Jose, CA, USA), and data were processed using FlowJo software (ver. 10; Tree Star, Ashland, OR, USA).

### 2.7. Adoptive Transfer Experiment of Septic Mice

Isolated CD4^+^ T cells from the WT mice were cultured with 2.5 µg/mL of eCIRP for 48 h in RPMI 1640 medium (Thermo Fisher Scientific), supplemented with 10% FBS, 100 U/mL Penicillin and 100 μg/mL Streptomycin, 1% glutamine and 50 μM 2-mercaptaethanol in a 37 °C incubator under humidified conditions containing 5% CO_2_, followed by stimulation with Cell Activation Cocktail (without Brefeldin A) (Cat. No.: 423302, Biolegend) for 4 h. Then, Th1-Treg cells, identified by surface CD25 and CXCR3 expression, were sorted by fluorescence-activated cell sorting (FACS) using a BD FACSAria (BD Biosciences). The purity of these sorted Th1-Treg cells consistently exceeded 90% according to FOXP3 and IFNγ expression. The 8–12-week-old male WT mice were subjected to CLP and injected intratracheally (i.t.) with 5 × 10^5^ cells of naïve CD4^+^ T cells or Th1-Treg cells. Cell viability was confirmed using trypan blue before intratracheal administration. The lungs were harvested 20 h after the surgery.

### 2.8. Real-Time Quantitative Reverse Transcription PCR

Total RNA was extracted from homogenized lung tissues using TRIzol reagent (Invitrogen, Thermo Fisher Scientific). cDNA was synthesized using murine leukemia virus transcriptase (Biosystems, Barcelona, Spain, Thermo Fisher Scientific). Quantitative Real-Time PCR was performed using gene-specific forward and reverse primers and SYBR Green PCR master mix (Biosystemes) using a StepOnePlus^TM^ real-time PCR machine (Biosystems). The sequences of the primers used are as follows: β-actin, (forward) CGTGAAAAGATGACCCAGATCA, (reverse) TGGTACGACCAGAGGCATACAG; IL-6, (forward) CCGGAGAGGAGACTTCACAG, (reverse) GGAAATTGGGGTAGGAAGGA; TNFα, (forward) AGACCCTCACACTCAGATCATCTTC, (reverse) TTGCTACGACGTGGGCTACA; IL-1β, (forward) CAGGATGAGGACATGAGCACC, (reverse) CTCTGCAGACTCAAACTCCAC; KC, (forward) GCTGGGATTCACCTCAAGAA, (reverse) ACAGGTGCCATCAGAGCAGT; and MIP2, (forward) CCAACCACCAGGCTACAGG, (reverse) GCGTCACACTCAAGCTCTG.

### 2.9. Lung Myeloperoxidase (MPO) Assay

A total of 50 to 100 mg of lung tissue was pulverized in liquid nitrogen and homogenized in KPO_4_ buffer containing 0.5% hexadecyltrimethylammonium bromide (Sigma-Aldrich, St. Louis, MO, USA) using a sonicator, with the samples kept on ice. The samples were subjected to 2 freeze/thaw cycles, and the supernatants were collected after centrifugation at 12,000× *g* for 15 min. The supernatants were diluted in a reaction solution containing *O*-dianisidine dihydrochloride (Sigma-Aldrich) and H_2_O_2_ (Thermo Fisher Scientific) as the substrate. Absorbance was measured at 460 nm, and MPO activity was calculated by determining the rate of change in optical density.

### 2.10. Lung Histology

Lung tissues were fixed in 10% formalin and embedded in paraffin. The paraffin-embedded lung tissue blocks were sectioned at 5 μm thickness and mounted on glass slides. The lung tissue sections were stained with hematoxylin and eosin (H&E) and examined using light microscopy. Lung injury scores were assessed using a scoring system established by the American Thoracic Society. Briefly, the scores were evaluated from 0 to 1 based on the presence of neutrophils in the alveolar and interstitial spaces, hyaline membranes, proteinaceous debris in the airspaces, and alveolar septal thickening [24].

### 2.11. Terminal Deoxynucleotidyl Transferase dUTP Nick End Labeling (TUNEL) Assay

To evaluate the presence of apoptotic cells in lung tissue, 5 μm lung tissue sections were stained using a commercially available TUNEL assay kit (In Situ Death Detection Kit, Roche Diagnostics, Indianapolis, IN, USA). Paraffin-embedded tissue sections were deparaffinized in xylene, digested with proteinase K, washed, and incubated with an enzyme solution containing terminal deoxynucleotidyl transferase enzyme and fluorescence-labeled nucleotides at 37 °C, according to the manufacturer’s protocol. Nuclei were counterstained with 4′,6-diamidino-2-phenylindole (DAPI, Vectashield Antifade Mounting Media, H-2000, Vector Laboratories, Newark, CA, USA). The slides were imaged using ZEISS LSM 900 confocal microscopy (ZEISS, Oberkochen, Germany). The TUNEL-positive cells in the lung tissue sections were quantified using ImageJ (version 1.54f; Fiji software, National Institute of Health, USA).

### 2.12. Lung Wet and Dry Ratio

Lung wet and dry weights were measured as previously described [25]. Briefly, the right lung was harvested and immediately weighed to determine the wet lung weight. The lung samples were then dried at 65 °C for 48 h and weighed again to obtain the dry weight. The wet-to-dry weight ratio was calculated as an indicator of pulmonary edema.

### 2.13. Statistical Analysis

Data analysis was performed using GraphPad Prism graphing and statistical software (ver 8.0.2; GraphPad Software, LLC, San Diego, CA, USA). The data presented in figures are expressed as mean ± SEM. Comparisons among multiple groups were performed using one-way analysis of variance (ANOVA) followed by Tukey’s multiple comparison test. Comparisons between two groups were performed using an unpaired two-tailed Student’s *t*-test. Survival rates were analyzed by the Kaplan–Meier estimator and compared with a Log-rank test. A *p*-value of <0.05 was considered statistically significant for comparisons between experimental groups.

## 3. Results

### 3.1. Th1-Treg Cells Are Increased in Septic Lungs

**Figure 1:**
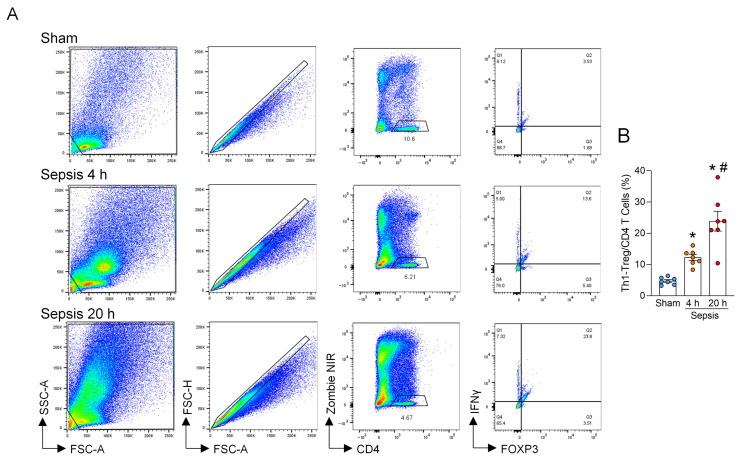
Th1-Treg cells are elevated in septic lungs. Sepsis was induced in WT mice using cecal ligation puncture (CLP). Lungs were collected at 4 h and 20 h after the surgery to examine the frequency of Th1-Treg cells by flow cytometry. (**A**) Representative gating strategy of flow cytometry plots for detecting Th1-Treg cells. (**B**) The frequency of Th1-Treg cells in sham mice, 4 h septic mice, and 20 h septic mice is shown. Experiments were performed 3 times, and all data were used for analysis. Data are expressed as mean  ±  SEM (n  =  7 samples/group) and compared by one-way ANOVA and Tukey’s multiple comparison test for multiple groups. * *p* < 0.05 vs. sham mice, ^#^ *p* < 0.05 vs. 4 h septic mice.

To investigate the involvement of Th1-Treg cells in the pathophysiology of sepsis, particularly in ALI, we first induced polymicrobial sepsis in WT mice by CLP and collected lungs at 4 and 20 h after surgery to assess the frequency of Th1-Treg cells in the lungs. The results demonstrated a significant, time-dependent increase in the frequency of Th1-Treg cells in septic lungs (Figure 1A,B). This data indicates that Th1-Treg cells are increased in the lungs during sepsis and may play a critical role in lung injury.

### 3.2. Th1-Treg Cells Are Induced by eCIRP in Sepsis

**Figure 2:**
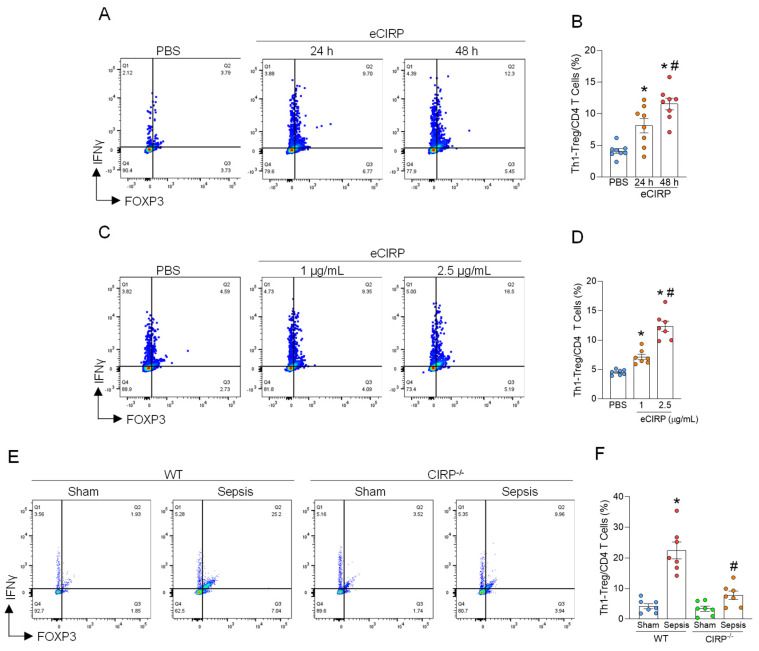
Th1-Treg cells are induced in septic lungs by eCIRP. CD4^+^ T cells from WT mice were cultured with (**A**,**B**) PBS or 2.5 μg/mL of eCIRP for 24 h or 48 h or with (**C**,**D**) PBS, 1.0 or 2.5 μg/mL of eCIRP for 48 h. The frequency of Th1-Treg cells of PBS-treated CD4^+^ T cells and eCIRP-treated CD4^+^ T cells was analyzed by flow cytometry. Experiments were performed 3 times, and all data were used for analysis. Data are expressed as mean  ±  SEM (n  =  7, 8 samples/group) and compared by one-way ANOVA and Tukey’s multiple comparison test for multiple groups. * *p* < 0.05 vs. PBS, ^#^ *p* < 0.05 vs. 24 h. * *p* < 0.05 vs. PBS, ^#^ *p* < 0.05 vs. 2.5 μg/mL of eCIRP. Sepsis was induced in WT and CIRP^−/−^ mice by cecal ligation puncture (CLP). Lungs were collected at 20 h after the surgery to examine the frequency of Th1-Treg cells. (**E**,**F**) The frequency of Th1-Treg cells in WT sham mice and WT septic mice, and CIRP^−/−^ sham mice and CIRP^−/−^ septic mice is shown. Experiments were performed 3 times, and all data were used for analysis. Data are expressed as mean  ±  SEM (n  =  7 samples/group) and compared by one-way ANOVA and Tukey’s multiple comparison test for multiple groups. * *p* < 0.05 vs. WT sham mice, ^#^ *p* < 0.05 vs. WT septic mice.

During sepsis, a significant increase in eCIRP in serum at 20 h after CLP was observed (Supplemental Figure S1). In previous studies, eCIRP has been shown to induce immune cell polarization [18]. As such, we first sought to determine the role of eCIRP on the induction of Th1-Treg cells. We cultured CD4^+^ T cells with different time courses of stimulation and different concentrations of eCIRP. We found that Th1-Treg cells were induced by eCIRP in a time- and dose-dependent manner (Figure 2A–D). Next, we subjected WT and CIRP^−/−^ mice to CLP and harvested the lungs at 20 h after surgery to determine the association between eCIRP and the increase in Th1-Treg cells in the septic lungs. While Th1-Treg cells were increased in the lungs of WT septic mice compared to the lungs of WT sham mice, this induction was significantly suppressed in the lungs of CIRP^−/−^ septic mice compared to those of WT septic mice (Figure 2E,F). These findings indicate that eCIRP directly induces Th1-Treg cells in septic lungs.

### 3.3. eCIRP Activates STAT1 and STAT5 via TLR4 to Induce Th1-Treg Cells

**Figure 3:**
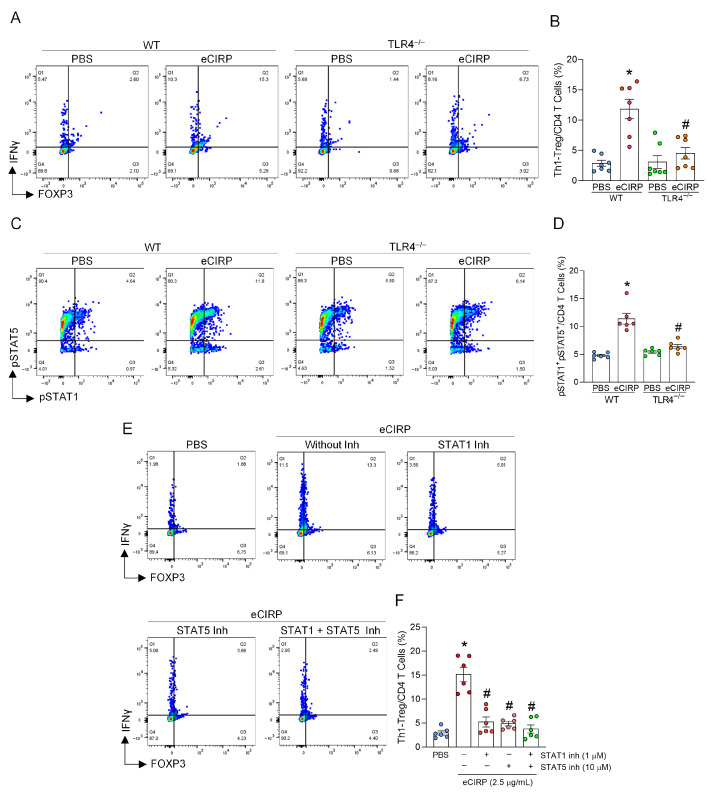
eCIRP activates STAT1 and STAT5 via TLR4 to induce Th1-Treg cells. (**A**,**B**) CD4^+^ T cells from WT and TLR4^−/−^ mice were cultured with PBS or 2.5 μg/mL of eCIRP for 48 h. The frequency of Th1-Treg cells of PBS-treated CD4^+^ T cells and eCIRP-treated CD4^+^ T cells was evaluated by flow cytometry. (**C**,**D**) CD4^+^ T cells from WT and TLR4^−/−^ mice were cultured with 2.5 μg/mL of eCIRP for 2 h. The frequency of pSTAT1^+^pSTAT5^+^ cells of PBS-treated CD4^+^ T cells and eCIRP-treated CD4^+^ T cells was evaluated by flow cytometry. Experiments were performed 2 times, and all data were used for analysis. Data are expressed as mean  ±  SEM (n  =  6, 7 samples/group) and compared by one-way ANOVA and Tukey’s multiple comparison test for multiple groups. * *p* < 0.05 vs. WT PBS, ^#^ *p* < 0.05 vs. WT eCIRP. (**E**,**F**) CD4^+^ T cells from WT mice were cultured with 2.5 μg/mL of eCIRP for 48 h in the presence and absence of 1 μM of STAT1 inhibitor and 10 μM of STAT5 inhibitor. The frequency of Th1-Treg cells was analyzed by flow cytometry. Experiments were performed 2 times, and all data were used for analysis. Data are expressed as mean  ±  SEM (n  =  6 samples/group) and compared by one-way ANOVA and Tukey’s multiple comparison test for multiple groups. * *p* < 0.05 vs. PBS, ^#^ *p* < 0.05 vs. eCIRP alone.

Next, we sought to elucidate the molecular mechanisms underlying the induction of Th1-Treg cells by eCIRP. As TLR4 is known as the main pattern recognition receptor (PRR) through which eCIRP facilitates cell polarization and inflammatory responses [18], we investigated whether TLR4 contributes to the induction of Th1-Treg cells by utilizing CD4^+^ T cells isolated from TLR4^−/−^ mice. eCIRP treatment significantly increased Th1-Treg cells compared to PBS treatment in WT CD4^+^ T cells, whereas this induction of Th1-Treg cells was significantly suppressed in eCIRP-treated CD4^+^ T cells isolated from TLR4^−/−^ mice (Figure 3A,B). These results indicate that TLR4 is the receptor mediating the induction of Th1-Treg cells by eCIRP. Next, we investigated the downstream signaling pathway of TLR4 in the induction of Th1-Treg cells. STAT1 is known as the inducer of Th1 polarization [26], while STAT5 promotes Foxp3 expression [27]. We examined the changes in the expression of pSTAT1 and pSTAT5 in CD4^+^ T cells after treating them with eCIRP. eCIRP significantly upregulated the frequency of pSTAT1^+^pSTAT5^+^ cells time dependently in WT CD4^+^ T cells (Supplemental Figure S2), whereas this upregulation was significantly reduced in TLR4^−/−^ CD4^+^ T cells (Figure 3C,D). Moreover, inhibitors of STAT1 and STAT5 significantly attenuated the induction of Th1-Treg cells by eCIRP (Figure 3E,F). Taken together, eCIRP induces Th1-Treg cells in CD4^+^ T cells through TLR4-mediated activation of STAT1 and STAT5.

### 3.4. Th1-Treg Cells Exacerbate Lung Inflammation in Sepsis

**Figure 4:**
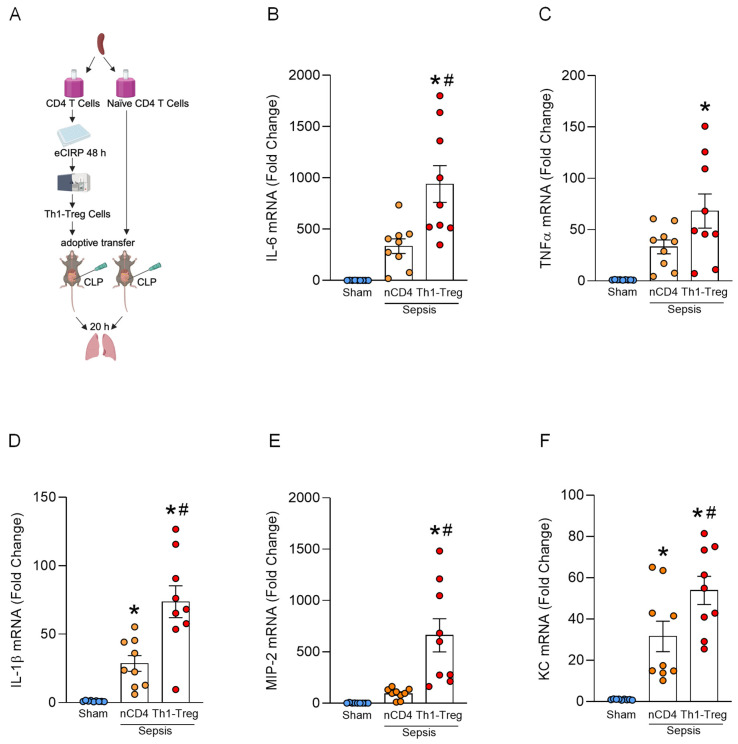
Th1-Treg cells exacerbate lung inflammation in sepsis. (**A**) CD4^+^ T cells were treated with 2.5 μg/mL of eCIRP for 48 h, followed by stimulation with Cell Activation Cocktail (without Brefeldin A). Th1-Treg cells were sorted by fluorescence-activated cell sorting following the gating strategy in Supplemental Figure S3. Naïve CD4^+^ T cells or Th1-Treg cells were adoptively transferred into septic mice via the trachea to collect lungs after 20 h. Created in BioRender. Lung tissue mRNA expressions of (**B**) IL-6, (**C**) TNFα, (**D**) IL-1β, (**E**) MIP-2, and (**F**) KC were measured by PCR. Experiments were performed 3 times, and all data were used for analysis. Data are expressed as mean  ±  SEM (n  =  9 samples/group) and compared by one-way ANOVA and Tukey’s multiple comparison test for multiple groups. * *p* < 0.05 vs. sham, ^#^ *p* < 0.05 vs. nCD4 (naïve CD4^+^) T cell-administered septic mice.

To examine the impact of Th1-Treg cells on lung inflammation in sepsis, we conducted an adoptive transfer experiment on septic mice (Figure 4A). We administered naïve CD4^+^ T cells or Th1-Treg cells intratracheally to septic mice and harvested the lungs at 20 h after the surgery. Then, we measured the expression of inflammatory cytokines and chemokines in the lungs, such as IL-6, TNFα, IL-1β, keratinocyte chemoattractant (KC), and macrophage inflammatory protein-2 (MIP-2). A significant increase in the expression of inflammatory cytokines and chemokines in lungs was observed in septic mice administered Th1-Treg cells compared to sham mice and septic mice administered naïve CD4^+^ T cells (Figure 4B–F). These findings indicate that Th1-Treg cells contribute to the aggravation of lung inflammation in sepsis.

### 3.5. Th1-Treg Cells Aggravate ALI and Mortality in Sepsis

**Figure 5:**
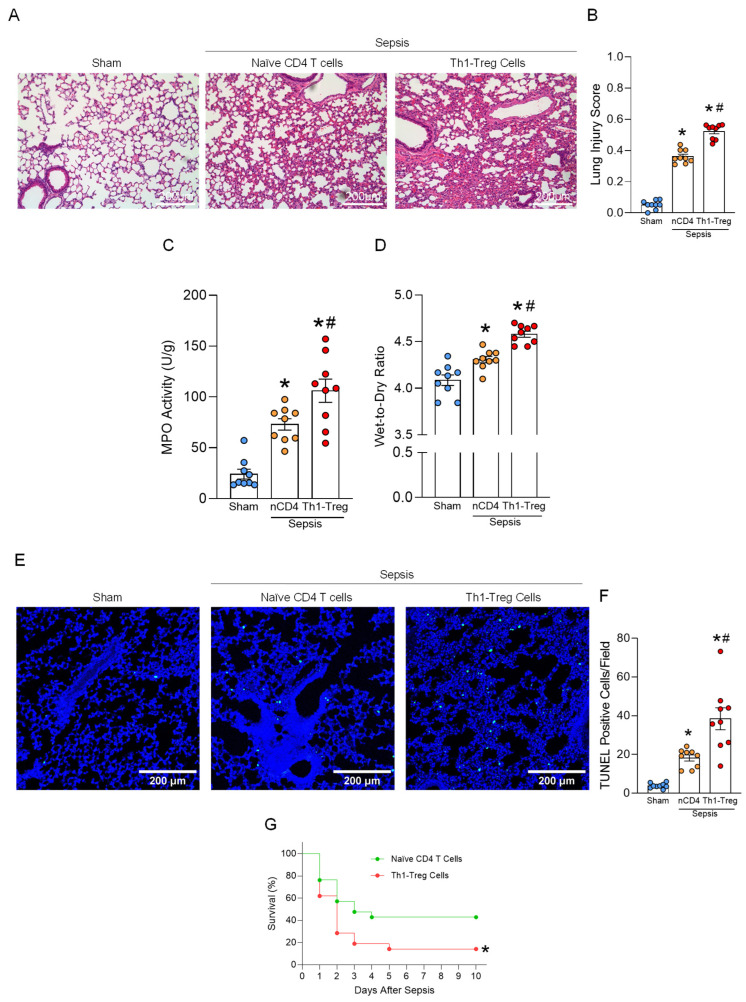
Th1-Treg cells aggravate lung injury and mortality in sepsis. Naïve CD4^+^ T cells or Th1-Treg cells were adoptively transferred to mice subjected to CLP-induced sepsis. The lungs were collected 20 h after the surgery. (**A**) Representative histological hematoxylin and eosin (H&E) images of lung tissues are shown. Scale bars: 200 μm. (**B**) Lung injury scores were calculated from 0 to 1 based on alveolar and interstitial neutrophil infiltration, hyalinization, protein filling in the airspaces, and wall thickening. (**C**) Myeloperoxidase (MPO) activity was assessed using colorimetric assays. Experiments were performed 3 times, and all data were used for analysis. (**D**) The lung wet-to-dry weight ratio was determined to evaluate pulmonary edema. Experiments were performed 4 times, and all data were used for analysis. (**E**) Representative images of TUNEL staining and (**F**) the number of TUNEL^+^ cells in lung tissues. Scale bars: 200 μm. Experiments were performed 3 times, and all data were used for analysis. Data are expressed as mean  ±  SEM (n  =  9 samples/group) and compared by one-way ANOVA and Tukey’s multiple comparison test for multiple groups. * *p* < 0.05 vs. sham, ^#^ *p* < 0.05 vs. nCD4 (naïve CD4^+^) T cell-administered septic mice. (**G**) Survival rates were analyzed by the Kaplan–Meier estimator using a log-rank test (n = 21/group). * *p* < 0.05 vs. naïve CD4^+^ T cell-administered septic mice. Experiments were performed 3 times, and all data were used for analysis.

**Figure 6:**
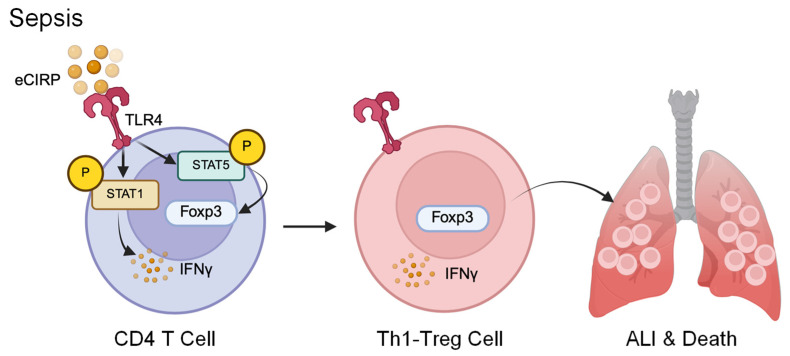
Finding summary. During sepsis, eCIRP signals through TLR4 to activate STAT1 and STAT5, driving the differentiation of pathogenic Th1-Treg cells. These cells accumulate in the lungs, exacerbate acute lung injury, and ultimately increase mortality in sepsis.

Next, we evaluated the contribution of Th1-Treg cells in lung injury and mortality in sepsis. Histological analysis revealed severe tissue injury in the lungs of septic mice. Th1-Treg cell adoptive transfer was found to significantly exacerbate this injury compared to naïve CD4^+^ T cells (Figure 5A,B). The lung MPO activity was significantly higher in septic mice administered Th1-Treg cells compared to sham mice and septic mice administered naïve CD4^+^ T cells (Figure 5C). Additionally, pulmonary edema was examined by measuring the lung wet-to-dry ratio, which demonstrated that Th1-Treg cells significantly exacerbated sepsis-induced pulmonary edema (Figure 5D). Cell death in lung tissues was assessed using the TUNEL assay, which revealed that Th1-Treg cells significantly contributed to an increase in apoptotic cells in lung tissues (Figure 5E,F). Finally, we investigated the effect of Th1-Treg cells on the survival of septic mice induced by CLP. Septic mice administered Th1-Treg cells had significantly higher mortality compared to septic mice administered naïve CD4^+^ T cells, and their survival rates were 14.2% and 42.9%, respectively. (Figure 5G). Together with the earlier findings, we have demonstrated that Th1-Treg cells are induced via the eCIRP-TLR4-STAT1/5 axis in septic lungs and aggravate ALI to worsen mortality in sepsis (Figure 6).

## 4. Discussion

In the present study, we demonstrate that Th1-Treg cells are significantly increased in the lungs during sepsis and that their accumulation contributes to exacerbated inflammation and enhanced cell death in the lungs and ultimately increased overall mortality. These findings suggest that Th1-Treg cells play a critical role in the dysregulated immune responses that drive lung injury during sepsis, rather than merely being bystanders. In addition, we reveal that, mechanistically, eCIRP, a new DAMP released during sepsis, induces the generation of Th1-Treg cells in septic lungs through TLR4-mediated activation of STAT1 and STAT5. These findings establish Th1-Treg cells as a pathogenic T cell subset in sepsis, linking their presence to organ dysfunction and adverse outcomes. To our knowledge, this is the first study to directly implicate these cells in septic lung injury pathogenesis.

The pathophysiology of sepsis is complex and includes inflammatory imbalance, immune system dysfunction, coagulopathy, and other pathophysiological processes that lead to multiple organ dysfunction [3]. The immune response to sepsis was classically considered to consist of early pro-inflammatory and later immunosuppressive phases [28]. In this regard, Th1 cells contribute to tissue injury through the excessive release of pro-inflammatory cytokines such as IFNγ in the state of hyperinflammation [29], whereas Treg cells contribute to susceptibility to infection by releasing anti-inflammatory cytokines such as IL-10 and TGFβ in the state of immunosuppression [30]. However, studies have shown that both pro- and anti-inflammatory mediators are elevated from the early stage in sepsis [31,32]. These observations suggest that pro-inflammatory and immunosuppressive responses are not strictly sequential but may occur in parallel during disease onset. Therefore, pro-inflammatory and immunosuppressive responses could coordinately contribute to the development of sepsis. Indeed, not only pro-inflammatory mediators, such as IFNγ and IL-6, but also anti-inflammatory cytokines, IL-10 and TGFβ, measured during the acute phase positively correlate with the severity of septic patients [32,33]. This indicates the importance of Th1-Treg cells, which may have both pro- and anti-inflammatory phenotypes. In general, pro-inflammatory cells are detrimental in inflammatory disorders but beneficial in cancer [34,35,36]. The multi-faceted property of Th1-Treg cells could be the reason why they exacerbate not only inflammatory disorders, such as sepsis and IBD, but also cancer [16,17].

In this study, eCIRP induced Th1-Treg cell differentiation through coordinated activation of STAT1 and STAT5 signaling downstream of TLR4 in CD4^+^ T cells. Pharmacological inhibition of either STAT1 or STAT5 equally abrogated the induction of Th1-Treg cells, indicating that both pathways are indispensable for this process. The requirement for both STAT pathways suggests that eCIRP-driven TLR4 activation initiates a STAT1-dependent Th1 program while concurrently promoting STAT5-mediated regulatory features that stabilize a hybrid Th1-Treg phenotype. These findings indicate that activation of both Th1 and Treg signaling within the same CD4^+^ T cells resulted in the induction of Th1-Treg cells. Prior studies suggest that Th1-Treg cells are generated from Treg cells by acquiring Th1 phenotypes under disease conditions [37]. Our study does not rule out the possibility that eCIRP transiently induces Treg cells, which then further transform to Th1-Treg cells. However, while STAT1 is known to be directly activated downstream of TLR4, STAT5 activation is generally mediated indirectly through secondary signaling [38,39]. Therefore, in the context of the eCIRP-TLR4 axis, it is possible that following Th1 polarization driven by STAT1 activation, subsequent STAT5 activation promotes the acquisition of regulatory T cell features in these cells. Determining the sequence of STAT1 and STAT5 activations would delineate the detailed mechanism of eCIRP-induced Th1-Treg induction. Moreover, further characterization of the stability and lineage commitment of Th1-Treg cells by assessing Helios and CD127 markers together with epigenetic Foxp3 stability would be informative.

We showed that Th1-Treg cells play an active role in shaping acute lung injury in sepsis. Although we did not determine the exact mechanism by which Th1-Treg cells exacerbate ALI—a limitation of this study—the molecular mechanisms of this tissue injury likely involve multiple aspects, considering their hybrid phenotype. It is plausible that Th1-Treg cells secrete IFNγ and IL-10/TGFβ, as they possess both Th1 and Treg phenotypes. Indeed, Th1-Treg cells have been reported to secrete IFNγ [16], and our data also demonstrated that IFNγ was elevated intracellularly in Th1-Treg cells. We have previously shown that IFNγ promotes neutrophil extracellular trap (NET) formation and exacerbates pulmonary inflammation and tissue injury during sepsis [40]. We evaluated ALI in sepsis using the ATS criteria, which include histologic evidence of lung injury, as demonstrated by a lung injury score; an altered alveolar–capillary barrier, as demonstrated by a lung wet-to-dry weight ratio; and an inflammatory response, as demonstrated by elevated levels of pro-inflammatory cytokines and MPO activity [24]. According to these criteria, we revealed that Th1-Treg cells aggravate ALI in sepsis. Collectively, Th1-Treg cells contribute to lung inflammation and tissue damage during sepsis, potentially through the combined release of pro-inflammatory cytokines, thereby amplifying immune-mediated pulmonary injury.

In our study, we specifically examined the role of eCIRP, a major DAMP in inflammation, in the induction of Th1-Treg cells. However, during sepsis, multiple DAMPs are elevated in the circulation and contribute to the overall immune responses [41]. Thus, it is possible that other DAMPs capable of activating TLR4 signaling may also participate in the generation of Th1-Treg cells. Our findings indicate that the activation of STAT1 and STAT5 leads to the induction of Th1-Treg cells. We investigated the involvement of STAT proteins in the induction process of Th1-Treg cells, given prior evidence that STAT1 is a key inducer of Th1 differentiation and STAT5 promotes Foxp3 expression [26,27]. In addition, other TLR4 downstream signaling pathways, such as NFκB, can be involved in the induction of Th1-Treg cells through secondary signaling. Janus kinase (JAK), another TLR4-mediated signaling, also has the potential to play a role in the induction of Th1-Treg cells, as JAK has been demonstrated to contribute to the activation of STAT proteins [42,43]. To determine the direct contribution of Th1-Treg cells to sepsis-induced lung injury, Th1-Treg cells were adoptively transferred into septic lungs. Nevertheless, the cellular origin of the Th1-Treg population under physiological conditions, whether derived from tissue-resident T cells or circulating T cells, was not examined. Future studies employing cell-tracking strategies will be necessary to clarify the origin of these Th1-Treg cells during sepsis.

Adoptive transfer via intratracheal administration has limitations, as it may not completely reflect the body’s natural immune system, potentially causing exaggerated lung tissue damage. However, Th1-Treg cells caused significantly more severe ALI compared to naïve CD4 T cells, even though both cells were injected intratracheally, indicating that Th1-Treg cells’ function is an independent factor in lung tissue damage. In the present study, we investigated the involvement of Th1-Treg cells in sepsis using a CLP model generated to achieve severity approximating the LD50. Although we did not assess less severe CLP models or LPS injection models, it is valuable to explore these conditions in future studies. We used only male mice to reduce internal heterogeneity and experimental variability, as female sex steroids can exert diverse immunomodulatory effects on both humoral and cell-mediated immune responses, potentially necessitating an inhumane number of animals to detect statistical significance [19]. However, studies using female mice would be valuable in the future. Although we used 8–12-week-old mice, which are considered young adults, future studies including mice of different ages would help to confirm the generality of our findings across a wider age range.

While the present study focused on the lung, which is a particularly vulnerable organ during sepsis, it is important to note that sepsis is a systemic syndrome affecting multiple organs, including the liver and kidneys. Investigating the impact of Th1-Treg cells in these additional organs may provide further insight into their contribution to sepsis-associated organ dysfunction. We used an infectious model of polymicrobial sepsis; however, Th1-Treg cells might be induced in other preclinical models, such as gut ischemia/reperfusion injury, which exhibit elevated levels of circulating eCIRP and are accompanied by organ dysfunction. The role of Th1-Treg cells in other preclinical models can be investigated further in the future.

In summary, the present study demonstrates that eCIRP induces the generation of Th1-Treg cells in the lungs during sepsis through activation of STAT1/5 proteins via TLR4. The accumulation of Th1-Treg cells in the lungs contributes to exacerbated inflammation, enhanced cell death, and ultimately increased mortality, thereby highlighting their pathogenic role in sepsis-induced ALI.

## Data Availability

The original contributions presented in this study are included in this article/Supplementary Materials. Further inquiries can be directed to the corresponding author.

## Supplementary Materials

The following supporting information can be downloaded at: https://www.mdpi.com/article/10.3390/cells15060521/s1, Figure S1: Serum was collected from sham mice and septic mice at 20 h after the surgery; Figure S2: CD4^+^ T cells from WT mice were cultured with PBS or 2.5 μg/mL of eCIRP treatment for 1 h or 2 h; Figure S3: Gating strategy for sorting Th1-Treg cells.

## Abbreviations

Th1: T helper 1, Treg: regulatory T, DAMP: damage-associated molecular pattern, eCIRP: extracellular cold-inducible RNA protein, TLR4: Toll-like receptor 4, STAT: signal transducer and activator of transcription, ALI: acute lung injury.
