# Supplementary material for: Pathologic Th1–Treg Cells Exacerbate Acute Lung Injury and Lethality in Sepsis
Source: Cells. 2026 Mar 14;15(6):521. doi: 10.3390/cells15060521 (PMC13024933; doi:10.3390/cells15060521)
Supplement: Supplementary file 1 [file cells-15-00521-s001.zip › cells-4153758-supplementary.pdf]

Supplemental Figures and Legends

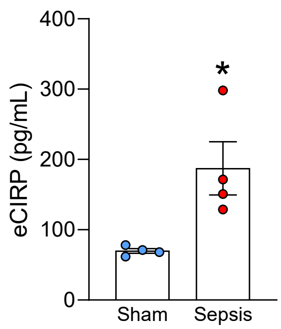

Supplemental Figure S1.

Serum was collected from sham mice and septic mice at 20 h after the surgery. Serum levels of eCIRP were shown. Data are expressed as mean ± SEM (n = 4 samples/group) and compared by unpaired two-tailed Student *t*-test. *p< 0.05 vs. sham mice.

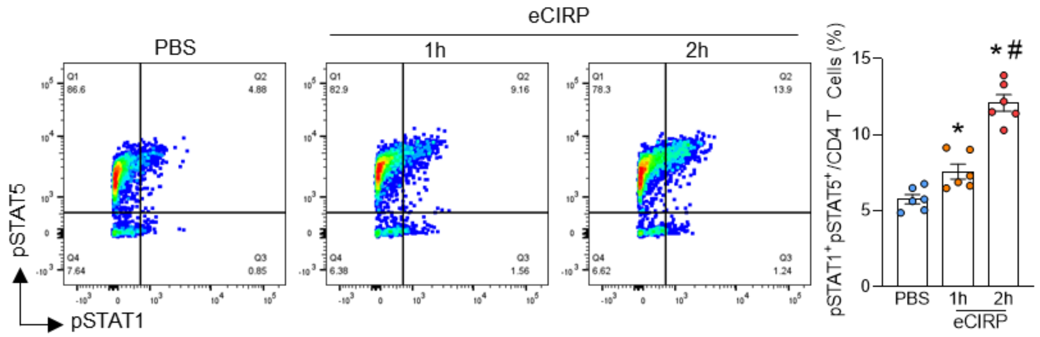

Supplemental Figure S2.

$CD4^+$ T cells from WT mice were cultured with PBS or 2.5 μg/mL of eCIRP treatment for 1 h or 2h. The frequency of $pSTAT1^+pSTAT5^+$ cells of PBS-treated $CD4^+$ T cells and eCIRP-treated $CD4^+$ T cells was evaluated by flow cytometry. Experiments were performed 2 times, and all data were used for analysis. Data are expressed as mean ± SEM (n = 6, 7 samples/group) and compared by one-way ANOVA and Tukey's multiple comparison test for multi groups. *p< 0.05 vs. PBS, $^{\#}$p< 0.05 vs. eCIRP 1 h.

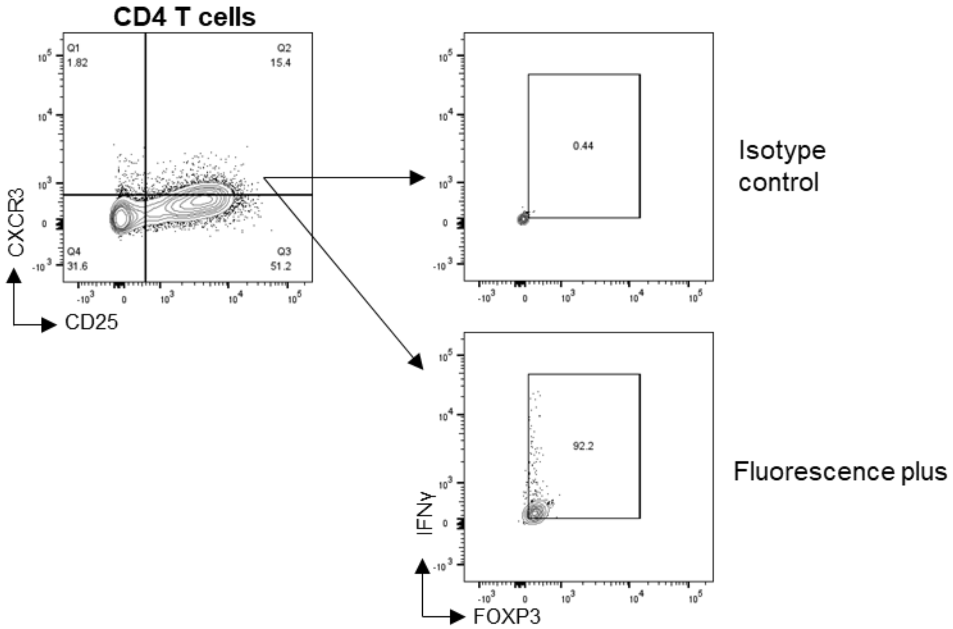

Supplemental Figure S3.

Gating strategy for sorting Th1-Treg cells. Th1-Treg cells were sorted based on their expression of CD25 and CXCR3. The expression of IFNγ and Foxp3 in these cells was confirmed using isotype controls.
